# Qualitative Needs Assessment study of Mental Health Service Networks

**DOI:** 10.1192/j.eurpsy.2025.2165

**Published:** 2025-08-26

**Authors:** I. Makivic, A. Žurga, A. Prijon, N. Ropret, M. Vinko

**Affiliations:** 1 Nacionalni inštitut za javno zdravje, Ljubljana, Slovenia

## Abstract

**Introduction:**

Assessing the needs within the field of mental health is crucial for the effective allocation of resources and the development of tailored interventions. A well-functioning network of services plays a vital role in addressing the complexities of mental health issues, which range from mild anxiety to severe psychiatric disorders. Understanding the local context and the diverse needs of the population can enhance the delivery of mental health services and improve overall community well-being.

**Objectives:**

The primary objective of this study was to assess the needs related to mental health services in two Slovenian regions. We aimed to identify the challenges faced by individuals with mental health issues, their families, and different professionals involved in their care. Furthermore, we sought to evaluate the existing service networks and the gaps in mental health support, highlighting the necessity for a coordinated approach in addressing these challenges. By understanding, the specific needs and different services available in both regions, we aim to inform policymakers and stakeholders about potential improvements and strategies for enhancing mental health service network.

**Methods:**

A semi-structured guided conversation was conducted with individuals and professionals working in mental health services. Coordinators that guided the conversation used specific questions. The data collected was analyzed to identify prevalent mental health issues within the local communities, potential solutions for the identified challenges, and the quality of collaboration among various stakeholders and services in the field of mental health.

**Results:**

Both regions reported significant mental health challenges, including high rates of anxiety, social isolation, and dual diagnoses among users. Common issues included long waiting times for services and stigma surrounding mental health. Service providers faced challenges like staff shortages, lack of training, insufficient time for treatment, and excessive administrative tasks. Both environments highlighted the need for improved inter-sectoral collaboration and community support programs. Systemic issues included lack of continuous funding, and inadequate living arrangements for people with mental health problems.

**Conclusions:**

The findings underscore the importance of a coordinated approach to mental health services that fosters collaboration among stakeholders. A robust service network is essential for addressing the diverse needs of individuals with mental health challenges. Future efforts should emphasize enhancing multisectoral collaboration, clarifying roles within the network, and involving families in care processes. By prioritizing these elements, we can create a more supportive environment for those affected by mental health issues and facilitate their recovery.

**Disclosure of Interest:**

None Declared

